# Characterization of Individuals with High-Frequency Artificial Tear Supplement Use

**DOI:** 10.3390/jcm14082694

**Published:** 2025-04-15

**Authors:** Wan-Lin Wu, Shu-Wen Chang

**Affiliations:** 1 Department of Ophthalmology, Far Eastern Memorial Hospital, No. 21, Section 2, Nanya S. Road, Banqiao District, New Taipei City 220, Taiwan; arielwu10101@gmail.com; 2 Department of Ophthalmology, National Taiwan University Hospital, 7 Chung-Shan S. Road, Taipei City 220, Taiwan

**Keywords:** high-frequency eye drop usage, dry eye disease, OSDI, SPEED, Schirmer score

## Abstract

**Objectives**: We aimed to investigate dry eye parameters as potential predisposing factors and estimate the prevalence of high-frequency topical eye drop usage. **Methods**: A total of 5594 dry eye patients treated between November 2015 and June 2022 were included. High users (*n* = 180) were those who applied at least one artificial tear drop per hour, whereas those who used artificial tears fewer than four times daily were classified as low users (*n* = 5414). Differences in self-reported symptoms (OSDI, SPEED questionnaires) and tear-related parameters, including severity of corneal staining (SPK), fluorescein tear-film break-up time (FTBUT), lipid layer thickness (LLT), number of expressible meibomian glands (MGE), meiboscale, and blink patterns, were assessed. Subsequent follow-up comprehensive dry eye assessments were performed at 3 months. **Results**: There was no difference in age or sex between high users and low users (*p* = 0.075 and 0.508, respectively). High users had significantly higher symptom scores (*p* < 0.001), more total blinks (*p* = 0.001), lower Schirmer scores (*p* < 0.001), higher SPK grades (*p* < 0.001), shorter FTBUT (*p* = 0.010), and higher limbal redness scores (*p* = 0.002). However, there were no differences in the LLT, MGE, or meiboscale. The compliance with follow-up examinations at 3 months was significantly greater for the high users (*p* < 0.001). Patients with OSDI scores > 40, SPEED scores > 12, Schirmer scores ≤ 3 mm, and higher compliance with follow-up examinations had odds ratios of 4.0, 3.3, 1.7, and 4.1, respectively, for being high users (95% confidence intervals = 2.8–5.8, 2.4–4.7, 1.2–2.3 and 2.7–5.2, respectively). Among the high users, reducing topical drops significantly decreased the SPEED and OSDI scores, except for the environmental trigger factor in the OSDI questionnaire. During long-term follow-up, 1.1% of low users and 15.4% of high users received cyclosporine treatment (odds ratio 16.4, *p* < 0.001). **Conclusions**: OSDI scores > 40, SPEED scores > 12, and Schirmer scores ≤ 3 mm were associated with high-frequency eye drop usage, which accounted for 3.2% of moderate to severe dry eye patients. Susceptibility to environmental triggers could represent hyperalgesia/allodynia in high users. High users have a higher need for cyclosporine treatment.

## 1. Introduction

Subjective symptoms are among the key diagnostic criteria for dry eye disease (DED) [1]. Pain in DED patients is a significant global concern due to its high prevalence and impact on work functioning. Improving pain management has become a critical priority across medical fields, including DED, to enhance the quality of care [2,3,4,5]. Acute nociceptive ocular surface pain is a temporary physiological response to tissue damage that diminishes over time with or without treatment [6,7,8]. Chronic ocular surface pain is a feeling of pain originating from the ocular surface that persists for more than 3 months and significantly interferes with daily activities [9]. Neuropathic patients with ocular surface pain frequently present with dryness, burning, aching, and tenderness. They are often diagnosed with DED, as dry eye symptom severity and persistence are associated with symptoms of neuropathic pain [4,5]. However, the ocular signs in these patients are usually insignificant.

The severity of subjective dry eye symptoms varies among individuals. Age [10], greater number of blinks [11,12], history of refractive surgery [13], subnormal sebum and/or denatured meibum from dermatochalasis skin [12], mixed aqueous and lipid deficient dry eye subtype [11], low ambient temperature [14], non-Sjogren’s syndrome [15], and hypersensitivity to wind and allodynia [4,5,16] are associated with higher subjective symptom scores.

Most DED patients with mild to moderate symptoms are generally treated by repeated instillations of artificial tears upon feelings of discomfort [8,17,18]. The recommended dosage regimen of artificial tears for the alleviation of DED symptoms is 4–6 instillations per day. However, more frequent instillations, e.g., hourly instillations, might be applied to relieve very severe symptoms, particularly reported eye pain. Nevertheless, elective topical medications including artificial tears, gels, and ointments have been reported to have a risk of affecting the ocular surface [19]. In addition, the presence of significant objective signs in patients necessitating hourly dosing for severe symptoms has not been demonstrated.

Medication adherence is related to patient characteristics, drug tolerance [20], and communication between physicians and patients [21]. In ophthalmology, compliance with medications is approximately 10.2 to 60% [21,22]. In contrast to medication compliance, over-the-counter accessibility leads to self-medication of artificial tears being a common path. However, self-medication can potentially lead to medication overuse and subsequently result in secondary disease. One common medication overuse disease is medication overuse headache [23,24], whose prevalence is approximately 1% [24]. The high prevalence of dry eye and the high availability of over-the-counter eye drops could lead to overuse, especially for those with more severe dry eye symptoms with or without hyperalgesia and/or allodynia. Preservatives in multidose formulations can cause cytotoxic damage to conjunctival and corneal epithelia, resulting in higher subjective symptom scores [25], limbal epithelial thinning [26], and corneal lesions [27]. However, the proportion of dry eye patients with medication overuse and the characteristics of these patients have not been reported. In this study, the subjective and objective dry eye parameters of patients who were experiencing medication overuse were analyzed. The proportion of patients experiencing medication overuse and the proposed temporary alternating patching as a treatment modality for symptom relief in severely symptomatic patients were also studied.

## 2. Materials and Methods

### 2.1. Patient Grouping

**Figure 1:**
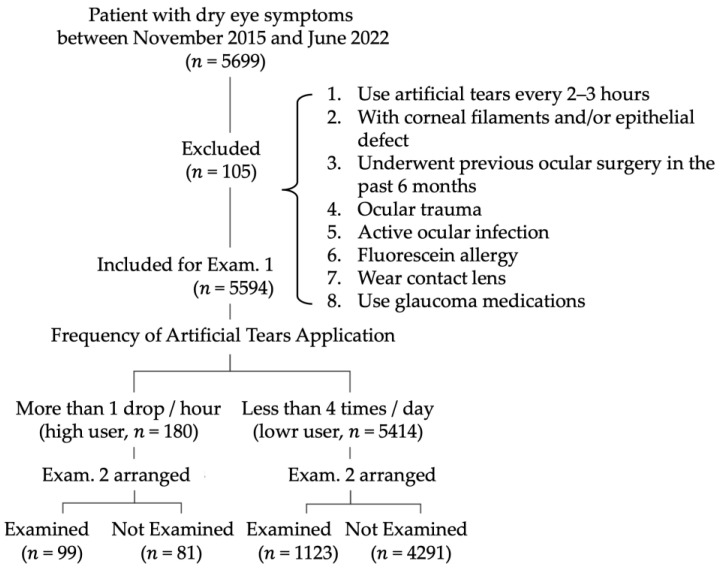
Patient inclusion/exclusion flow chart.

The study protocol adhered to the Declaration of Helsinki and was approved by the Institutional Review Board. Patient consent was waived because this was a retrospective study involving low-risk patients. A total of 5699 patients were included, all of whom visited the outpatient clinic of one tertiary hospital for dry eye evaluation between November 2015 and June 2022. The included patients (*n* = 5594) were aged 20 years or older. The exclusion criteria are summarized in Figure 1 (*n* = 105). Within the cohort, 180 patients were identified as high users, defined as those using artificial tears at least once per hour, whereas 5414 patients were classified as low users, defined as those using artificial tears no more than four times daily (Figure 1). To minimize the confounding effects of topical gels/ointments, the treatments were discontinued for at least 1 day before comprehensive dry eye examinations [12], which were conducted within 1 week of visiting our dry eye clinic. The high users were asked to limit artificial tears to less than 4 times per day. Those high users who experienced intolerable eye pain were instructed to patch the lid closed in one eye alternately for 4 h for a duration of 1 week. All patients were instructed to perform lid care, including lid hygiene, warm compresses, and blink exercises, after comprehensive dry eye examinations. Topical 0.1% flurormetholone and artificial tears were prescribed four times per day for 3 months. The intraocular pressure was measured before inclusion and every month. Flurormetholone was discontinued once an intraocular pressure higher than 21 mmHg was found. Follow-up comprehensive dry eye examinations were scheduled at 3 months. The presence of eye conditions (e.g., pseudophakia, keratoconus, and chemical burns) or systemic conditions such as sicca syndrome, rheumatoid arthritis, Stevens–Johnson syndrome, systemic lupus erythematosus, diabetes mellitus, asthma, menopause/hysterectomy/oophorectomy was also recorded.

### 2.2. Examination Protocol

Following the completion of symptom questionnaires, patients underwent evaluations conducted by well-trained technicians, including assessments of lipid thickness, blink number, meibomian gland function, ocular surface redness, and aqueous tear measurements. An ophthalmologist subsequently assessed the corneal staining and fluorescein tear break-up time (FTBUT). The specifics of the examination are outlined below.

### 2.3. Subjective Symptoms

Subjective symptoms were evaluated via the Standardized Patient Evaluation of Eye Dryness (SPEED) and Ocular Surface Disease Index (OSDI) questionnaires. The frequency and severity score derived from the SPEED questionnaire, along with the three individual subtotal scores from the OSDI questionnaire (subtotal A: symptom frequency; subtotal B: activity limitation frequency; subtotal C: environmental trigger discomfort frequency), were analyzed.

### 2.4. Grading of Superficial Punctate Keratitis (SPK) and Measurement of the FTBUT

Fluorescein staining (fluorescein sodium ophthalmic stripes, U.S.P., Enkelihoito, China) was performed, and the severity of SPK was graded by an ophthalmologist from 0 to 4 according to the Oxford scheme. Afterwards, the FTBUT was measured three times and averaged for subsequent analysis, as previously described [11,28].

### 2.5. Lipid Layer Thickness (LLT) and Blinks

The average LLT was quantified via a LipiView II interferometer (Johnson & Johnson Vision, Jacksonville, FL, USA). The total number of blinks during the 20 s observation period was documented. The incomplete blink rate was calculated by dividing the number of incomplete blinks by the total number of blinks and then multiplying by 100%.

### 2.6. Number of Expressible Meibomian Glands (MGEs)

The number of MGEs was assessed using a handheld Meibomian Gland Evaluator™ (TearScience® Inc., Morrisville, NC, USA) through slit-lamp biomicroscopy [28]. The cumulative count of MGEs from both the upper and lower eyelids was used for the subsequent analysis.

### 2.7. Meiboscale

The meibography was conducted using a LipiView II interferometer (Johnson & Johnson Vision, Jacksonville, FL, USA). One well-trained examiner graded the meibomian gland dropout as meiboscale as previously described [11,28]. The meiboscale grades of the upper and lower eyelids were determined separately.

### 2.8. Aqueous Tear Secretion Evaluation

The amount of basal aqueous tears was evaluated with a Schirmer test I with anesthetics and a standard 35 mm × 5 mm tear test strip (Eagle Vision, Katena Products, Memphis, TN, USA) for 5 min.

### 2.9. Ocular Surface Redness

The severity of conjunctival and limbal redness was graded according to the Efron grading scale for contact lens complications [29] by a well-trained technician according to the Lipiview II external eye photographs.

### 2.10. Compliance

To evaluate whether compliance with dry eye treatment predisposes patients to medication overuse, patient compliance was evaluated. Patients who underwent follow-up examination by the same ophthalmologist and technician after the scheduled examination at 3 months were considered compliant, whereas those who were lost to follow-up were not compliant. The compliance rate was compared between high users and low users.

### 2.11. Dry Eye Subtyping

To investigate whether tear-film components contribute to increased artificial tear usage, patients were categorized into four groups on the basis of their Schirmer scores and LLT, as previously outlined: Type 1, Schirmer > 5 mm, LLT > 60 nm; Type 2, Schirmer > 5 mm, LLT ≤ 60 nm; Type 3, Schirmer ≤ 5 mm, LLT ≤ 60 nm; and Type 4, Schirmer ≤ 5 mm, LLT > 60 nm [11].

### 2.12. Long-Term Dry Eye Treatment and Follow-Up

After Examination 2, patients continued their individualized dry eye treatment and were followed up at the dry eye clinic. Their use of medications and artificial tears was recorded in the electronic medical record system for analysis. To be eligible for CsA coverage under a healthcare plan, patients must meet the National Health Institute reimbursement criteria for dry eye severity: (1) a Schirmer test result of ≤5 mm/5 min; (2) a TBUT of ≤5 s; (3) the presence of superficial punctate keratitis (SPK) at grade 3 or higher on the Oxford scale or other signs such as conjunctival congestion, filamentary keratitis, corneal epithelial defect (with or without ulceration), symblepharon, or corneal keratinization; and (4) no improvement following prior anti-inflammatory treatments, including topical steroids, punctal occlusion, and artificial tears. Cyclosporine was prescribed only when conventional dry eye treatments failed to provide optimal improvement, as per established national health insurance guidelines [28]. These criteria were consistent across both groups.

### 2.13. Statistical Analysis

For each patient, only data from the right eye were included in the analysis. Statistical analysis was performed using SPSS v.20.0 (IBM SPSS Statistics for Windows, IBM Corp., Armonk, New York, NY, USA). The Kolmogorov–Smirnov test was used to assess the normality of the numerical variables. The comparison of numerical variables, which included SPEED/OSDI questionnaire scores, LLT, Schirmer test results, meiboscale grades, number of MGEs, and total/incomplete blinks between the high users and low users, was performed using the Mann–Whitney U test. Descriptive findings are presented as the median and interquartile range (IQR). After univariate analysis, multivariate logistic regression analysis was conducted to explore the likelihood of becoming high users and the predictors. The chi-square test was conducted to examine the differences in the distributions of sex, compliance, dry eye subtype, and presence of systemic conditions and/or eye conditions. Changes in parameters before and after treatment were assessed using the Friedman test. *p*-values less than 0.05 were considered to indicate statistically significant results.

## 3. Results

### 3.1. Differences in Demographics and Dry Eye Parameters Between Low Users and High Users

**Figure 2:**
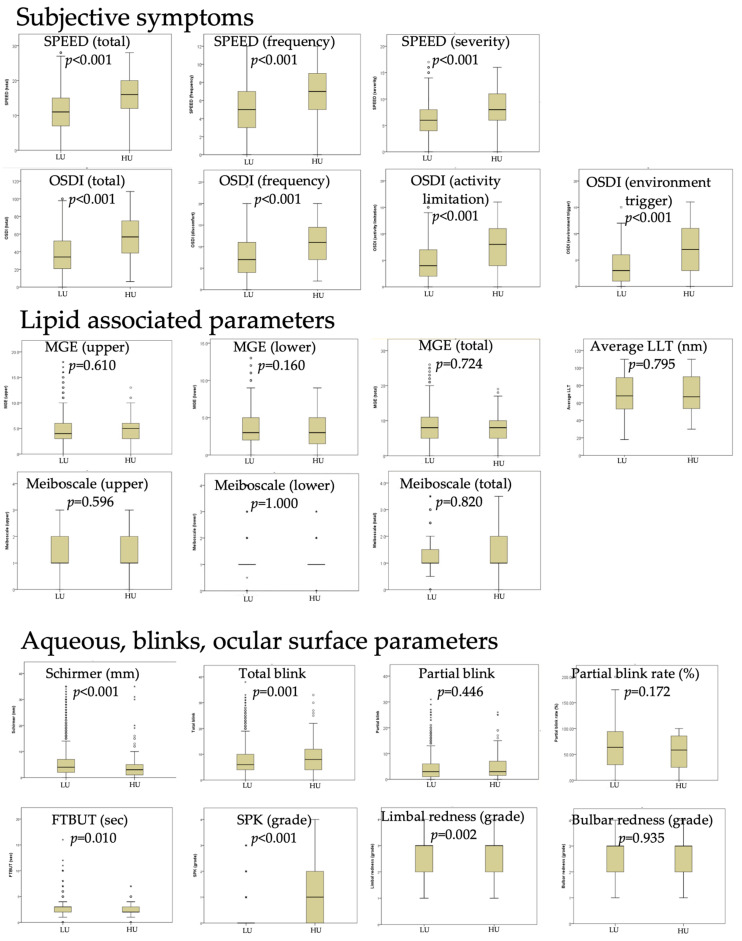
Comparison of baseline characteristics between high users (*n* = 180) and low users (*n* = 5414). LLT: average lipid layer thickness; MGE: number of expressible meibomian gland expression; FTBUT: fluorescein tear-film break-up time; SPK: superficial punctate keratitis. *p*: statistical significance examined by the Mann–Whitney U test. *: extreme outliers, more than three times the IQR away from the quartiles; 。: mild outliers, between 1.5 and 3 times the IQR away from the quartiles.

There were 3985 females and 1609 males enrolled, with a female predominance of 74.7% (3985/5594). Females accounted for 72.5% and 74.8% of the high users and low users, respectively. The low users and high users were aged 57.0 (21.0) and 54.0 (18.8) years, respectively. There was no significant difference in sex distribution or age between high users and low users (*p* = 0.508 and 0.075, respectively). Patients in the high-user group had significantly higher SPEED/OSDI scores than those in the low-user group (Figure 2). With an OSDI score > 40, the odds ratio of becoming high users was 4.0 (95% CI = 2.8, 5.8) compared with those with an OSDI score ≤ 40. With a SPEED score > 12, the odds ratio of becoming high users was 3.3 (95% CI = 2.4, 4.7) compared with those with an OSDI score ≤ 12.

The high users also had lower Schirmer scores (*p* < 0.001), shorter FTBUTs (*p* = 0.010), higher SPK grades (*p* < 0.001), and more limbal redness (*p* = 0.002) than the low users. With a Schirmer score ≤ 3 mm, the odds ratio of becoming a high user was 1.7 (95% CI = 1.2, 2.3) compared with those with a Schirmer score > 3 mm. The high users also had more total blinks. However, there was no significant difference in lipid-related parameters, including LLT, MGE, and meiboscale. High users had a greater prevalence of rheumatoid arthritis (*p* = 0.038) and sicca syndrome (*p* < 0.001). However, there was no difference in the prevalence of other systemic conditions such as systemic lupus erythematosus, diabetes mellitus, asthma, Steven–Johnson syndrome, menopause/hysterectomy/oophorectomy, or other eye conditions (*p* > 0.05 for all).

### 3.2. Distribution of Dry Eye Subtypes

**Figure 3:**
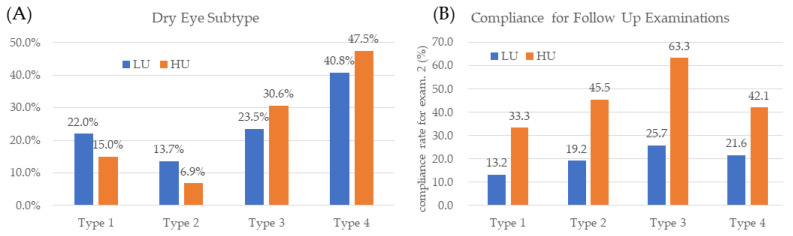
Distribution of dry eye subtypes of high users and low users. (**A**) There were significantly more Type 3 and 4 patients in the high-user group (chi-square *p* = 0.002). (**B**) The compliance rate was significantly higher in high users (chi-square test *p* < 0.001). LU: low user; HU: high user.

Patients with Type 3 or 4 dry eyes had a Schirmer ≤ 5 mm and were thus considered to have aqueous-deficient dry eyes. Types 2 and 3 had an LLT ≤ 60 nm and were thus considered to have evaporative dry eyes. There was a greater proportion of aqueous-deficient dry eyes among the high users than among the low users (*p* = 0.002) (Figure 3A).

### 3.3. Compliance for Examination 2

There were 99 high users and 1123 low users who completed follow-up examinations at 3 months (Figure 1). The compliance rate for follow-up examinations was significantly greater for high users than for low users (55.0% and 20.7% for high users and low users, respectively; *p* < 0.001) (Figure 3B). In the low-user group, patients compliant with Examination 2 had higher SPEED/OSDI scores (*p* < 0.05 for all parameters), thinner LLTs (*p* = 0.003), less MGE (*p* = 0.004), higher meiboscale grades (*p* = 0.001), lower Schirmer scores (*p* = 0.002), and more total blinks (*p* = 0.035). Compared with low users, high users were more compliant, with an odds ratio of 4.1 (95% CI = 2.9, 5.6). In high users, there was no significant difference in any of the subjective symptoms or objective parameters, whether they were compliant with Examination 2 or not. The multiple logistic regression models are shown in Table 1. These results suggest that increases in OSDI, SPEED, SPK grade, and bulbar redness are associated with increased odds of becoming high users. Lower LLT, Schirmer scores are also associated with increased odds of becoming high users.

### 3.4. Effects of Lid Care and Reducing Topical Eye Drop Application

**Figure 4:**
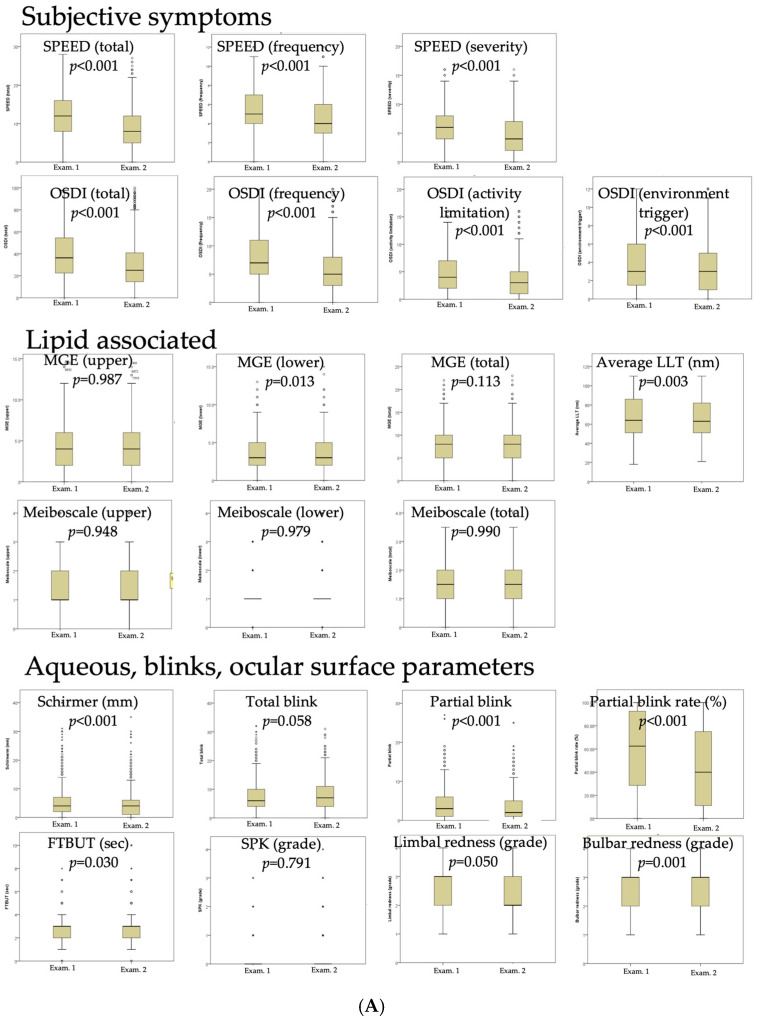

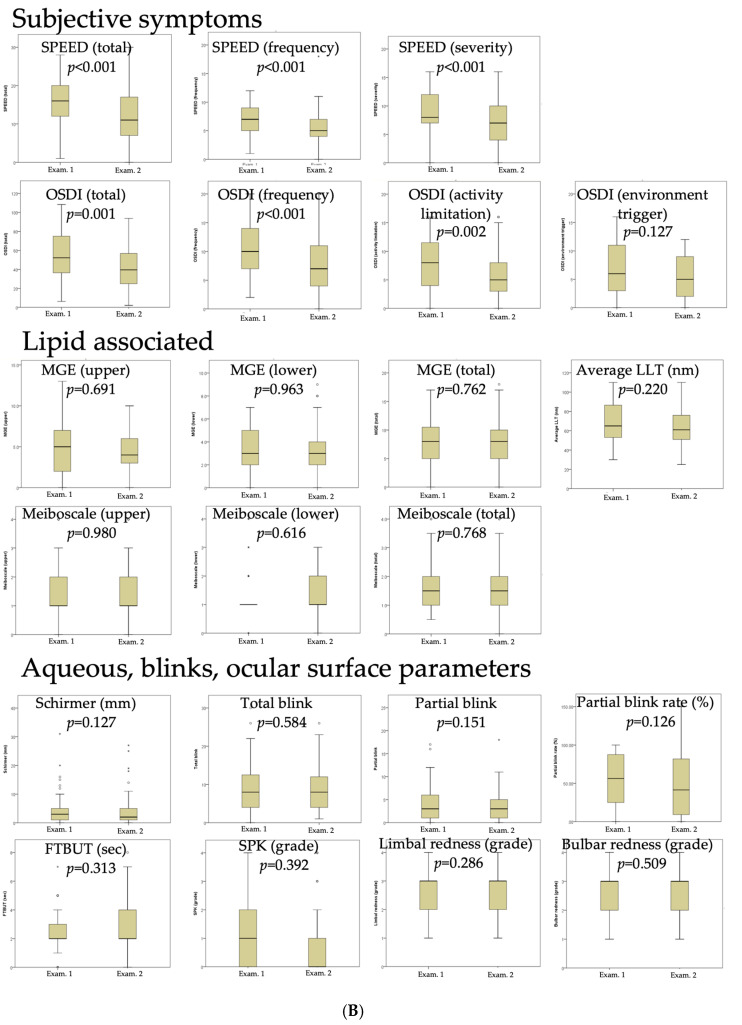
(**A**) Low users (*n* = 1123). Comparison of dry eye parameters between the 2 examinations of low users (**A**) and high users (**B**). (**B**) High users (*n* = 99). LLT: average lipid layer thickness; MGE: number of expressible meibomian gland expression; FTBUT: fluorescein tear-film break-up time; SPK: superficial punctate keratitis. *: extreme outliers, more than three times the IQR away from the quartiles; 。: mild outliers, between 1.5 and 3 times the IQR away from the quartiles.

For low users, lid hygiene, topical steroids, and artificial tear application significantly decreased SPEED and OSDI scores (Figure 4A). There was a significant decrease in the Schirmer score and in the blink-related parameters, including the number of partial blinks and the partial blink rate. However, there was no change in the total number of blinks. There was also an improvement in FTBUT and ocular redness. Among the high users, lid care, topical steroids, and reducing topical artificial tears significantly decreased the SPEED and OSDI scores (Figure 4B), except for the environmental trigger factor in the OSDI questionnaire. In addition, there was no change in the Schirmer score, lipid-related parameters, blink patterns, FTBUT, SPK grades, or ocular redness.

**Figure 5:**
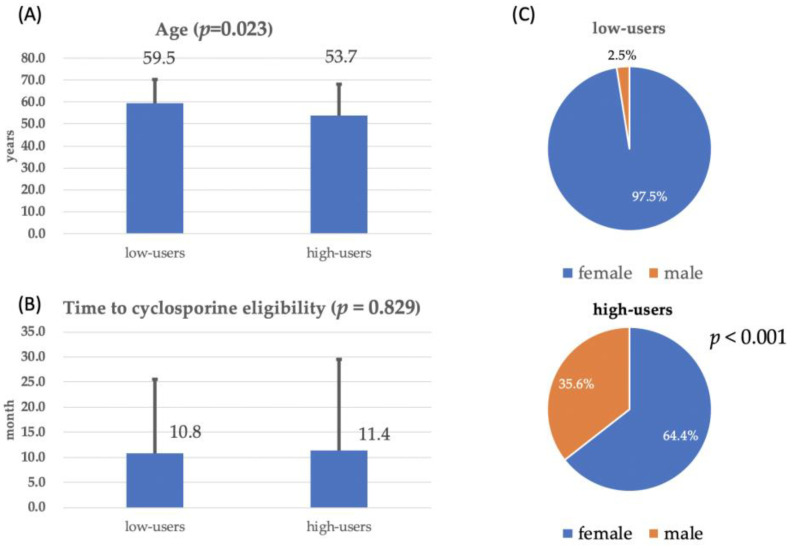
Comparison of age at the time of cyclosporine eligibility (**A**), time to cyclosporine eligibility (**B**), and sex distribution (**C**).

During the follow-up period, 1.1% of low users qualified for cyclosporine treatment under our national healthcare policy [28], compared to a significantly higher proportion of high users (15.4%, odds ratio: 16.4, *p* < 0.001). At the time of cyclosporine eligibility, high users were significantly younger (*p* = 0.023) and showed less pronounced female dominance (*p* < 0.001) (Figure 5). However, there was no significant difference in the time to cyclosporine eligibility between the two groups (*p* = 0.829).

## 4. Discussion

The 5594 enrolled patients had a median OSDI of 35.0 and a median TBUT of 3.0 s, indicating that most of the included patients had moderate to severe short BUT-type dry eyes [30]. Patients with short BUT dry eyes are hypersensitive to corneal pain [16], which may explain the median OSDI score of 34.1 and SPEED score of 11.0 observed in low users at their first visit to the dry eye clinic. In contrast, high users presented significantly higher symptom scores, with an OSDI score of 22.7 and a SPEED score of 5.0 higher than low users (Figure 2). These differences likely reflect medication overuse-related discomfort combined with hyperalgesia and/or allodynia in high users.

We propose that high users exhibited underlying allodynia, perpetuated by excessive artificial tear use as an environmental trigger. This behavior created a vicious cycle of ocular symptoms and frequent tear application for temporary relief. To disrupt this cycle, patients were advised to limit artificial tear use to fewer than four times daily and to patch their lids closed to reduce blink-related friction. While these interventions successfully halted the cycle, the underlying allodynia—manifested as heightened sensitivity to environmental triggers—persisted.

Dry eye treatment significantly improved subjective symptoms in both low users and high users (Figure 4). However, environmental trigger factors remained unresolved in high users, as reflected in their higher median OSDI and SPEED scores at Examination 2. Specifically, there was a 14.6-point difference in the OSDI score and a 3.0-point difference in the SPEED score between the two groups (Figure 4B). These differences are clinically meaningful, as an OSDI reduction of ≥13 points is considered the threshold for significant improvement [31]. By 3 months, overuse-related discomfort in high users should have been minimized due to the limitation of eye drop applications. The persistence of symptoms likely reflects sensory dysfunction, such as hyperalgesia or allodynia, rather than ongoing ocular surface abnormalities. Allodynia, commonly associated with dry eye disease, causes heightened sensitivity to environmental factors like wind and light [5]. The continued presence of environmental triggering symptoms (Figure 4B) suggests the involvement of neuropathic pain mechanisms in high users.

High users also had a greater proportion of Type 3 and 4 patients compared to low users (Figure 3A), consistent with their lower median Schirmer scores and higher symptom scores (Figure 2). Reduced lubrication due to lower Schirmer scores increases friction-related discomfort [32,33], which may predispose patients to frequent use of topical medications or artificial tears. For example, low users had a median Schirmer score of 4.0 and a median OSDI score of 54.1, whereas high users had a lower median Schirmer score of 3.0 and a higher median OSDI score of 56.8. Many high users, desperate to alleviate severe eye pain, were instructed to apply eye drops more frequently. This frequent self-medication behavior, often involving eye drops with preservatives, may exacerbate dry eye symptoms by inducing irritation and inflammation on the ocular surface [25,26,27].

The vicious cycle of medication overuse occurs when patients rely excessively on eye drops to relieve symptoms without addressing the underlying causes of dry eyes. Compliance behavior leads some patients to apply drops every 5 min to an hour. High users represented self-medication behaviors driven by a severe self-perception of illness and subjective symptoms. Patients and healthcare providers should recognize that frequent or prolonged use of eye drops, particularly those containing preservatives, can aggravate dry eye symptoms and perpetuate the cycle of discomfort.

Compliance with dry eye treatment is usually low and varies among studies. Only 10.2% of artificial tear users instill at the specified frequency [34]. In this study, low users typically visit at least one ophthalmologist and/or obtain several anti-inflammation and/or anti-infective medications/lubricants from various sources before visiting the dry eye center. Most of these patients cannot accurately describe the names of the eye drops used. To evaluate dry eye treatment effects, a series of comprehensive dry eye examinations were arranged 3 months after the patient conducted lid hygiene and started medications. In the hopes of alleviating the illness with the help of caregivers, it is reasonable that patients with more severe subjective symptoms are more compliant with follow-up examinations. These patients had higher SPEED/OSDI scores, thinner LLTs, less MGE, higher meiboscale grades, lower Schirmer scores, and more total blinks. Compared with low users, high users were even more compliant with the follow-up examinations. The rate for attending the follow-up examination was 20.7% for low users and 55.0% for high users. These patients had an odds ratio of 4.1 for becoming high users. High users had even higher subjective OSDI/SPEED scores. These results suggest that patients with pre-existing hyperalgesia/allodynia use more medications and/or artificial tears when they experience dry eye discomfort, potentially increasing their susceptibility to medication overuse. In addition, a great proportion of them apply anti-inflammation/anti-infective medications to substitute lubricants. This preservative/medication-induced keratopathy could be alleviated by stopping the offending medication while the pre-existing hyperalgesia/allodynia remained.

Blinking induces friction by inducing mechanical stimulation of the ocular surface, which is a consequence of increased symptoms of dryness and discomfort [35]. In addition, the number of blinks is associated with dry eye symptoms [11,12]. Although the number of blinks is not a direct ocular surface sign of dry eyes, it could be considered an indirect sign of dry eye severity.

In this study, 180 high users accounted for 3.2% of the 5594 newly enrolled dry eye patients. This is higher than the 1% rate of medication overuse for headaches [24]. This could have resulted from the high incidence of dry eye and the high availability of over-the-counter eye drops. Medication overuse can be managed by medication withdrawal and the initiation of prophylaxis [24]. In the dry eye clinic, limiting topical artificial tears to less than 4 times per day results in the withdrawal of the offending chemicals, and alternating patching reduces blink-related friction-related discomfort. Combining both approaches significantly alleviated subjective symptoms within 1 week in most patients, although there were no significant changes in other dry eye parameters at the 3-month follow-up examination.

Cyclosporine is an effective treatment for dry eyes; however, meta-analysis evidence suggests its efficacy may decrease when combined with artificial tears [36]. In Taiwan, the healthcare system subsidizes cyclosporine for severe dry eye cases that are unresponsive to traditional treatments, with eligibility reassessed every six months for continued reimbursement [28]—a process that helps control healthcare expenditures. These criteria were consistent across both groups. The lack of a significant difference in the time to cyclosporine eligibility (*p* = 0.829) is likely due to Taiwan’s standardized reimbursement policy. Since eligibility requires prior failure of conventional treatments and is reassessed every six months, both high and low users followed a similar treatment timeline, limiting variability. Although high users had more severe symptoms, cyclosporine approval is based on objective clinical criteria rather than symptom severity alone. Additionally, factors like ocular surface inflammation and tear-film instability influence disease progression, making eligibility timing less dependent on user classification. These factors, combined with the structured treatment protocol, likely explain the observed lack of difference between the two groups.

In this study, only 1.1% of low users required cyclosporine treatment, despite having a median OSDI score of 34.1 and SPEED score of 11.0, indicating that most had moderate to severe dry eye symptoms. This study identified key risk factors for high-frequency artificial tear use, including OSDI scores > 40, SPEED scores > 12, and Schirmer scores ≤ 3 mm. Patients meeting these criteria had a significantly higher risk of high-frequency eye drop use and were 16.4 times more likely to require cyclosporine treatment. Based on our results, we suggest that patients with an OSDI score > 40, a SPEED score > 12, and a Schirmer score ≤ 3 mm be considered for earlier evaluation for cyclosporine treatment, as they are at significantly higher risk of high-frequency artificial tear use and may have an underlying inflammatory component driving their symptoms. Incorporating these parameters into clinical decision-making could help identify patients who may benefit from earlier intervention, potentially improving symptom management and reducing reliance on excessive artificial tear use. Future studies should further explore how integrating these criteria into treatment guidelines impacts long-term patient outcomes.

At the time of cyclosporine eligibility, high users were significantly younger and showed a less pronounced female predominance. Since the National Health Institute’s reimbursement criteria for cyclosporine were based on objective signs and applied equally to both high and low users, this suggests that younger patients with significant dry eye signs are more likely to require long-term cyclosporine treatment. While younger patients may have fewer age-related dry eye risk factors, their lifestyle choices could contribute to significant ocular surface inflammation. In this group, inflammation may be more influenced by environmental factors, such as prolonged screen time and exposure to pollutants. Both men and women experience reduced blink rates and increased tear evaporation due to extended screen use, which may explain the weaker female predominance among younger cyclosporine users.

The strength of this study is that a relatively large number of high users who used artificial tears at more than one drop per hour were included. Some of the participants used one drop every 5 min. With a relatively large number of dry eye patients, this study was thus able to delineate risk factors leading to medication overuse. Awareness and early recognition of these predisposing factors will facilitate DED management for both ophthalmologists and non-ophthalmologists. One limitation of this study is that the corneal nerve was not directly evaluated by confocal microscopy, and the morphological associations with hyperalgesia and allodynia could not be confirmed. Further studies to determine the presence of microneuroma, alterations in dendritic cell density, and alterations in corneal nerve parameters could strengthen the conclusions of this study [37,38]. Another limitation is that prior anti-inflammation and/or anti-infective medications/lubricants used could not be analyzed. The high users typically visit at least three ophthalmologists and/or obtain several medications/lubricants from various sources. Most of them cannot accurately describe the names and frequency of eye drops used, rendering a relevant systematic analysis impossible. This is in concordance with the fact that only 15.5% of completed clinical trial registries were published due to the difficulty in objectively evaluating the efficacy and safety of topical lubricants [19]. A third limitation is that variables such as humidity, temperature, air quality, and screen exposure, which can influence dry eye symptoms, were not strictly controlled in this retrospective study. However, our findings reflect real-world conditions. Psychological factors, such as anxiety and depression, can influence dry eye symptoms and treatment adherence. Future research incorporating psychological evaluations could provide a more comprehensive understanding of their role in dry eye disease. In addition, future research with long-term follow-ups will be essential to better understand the chronicity of dry eye symptoms and the durability of treatment outcomes.

## 5. Conclusions

High eye drop application rates accounted for 3.2% of the moderate to severe dry eye patients. OSDI scores > 40, SPEED scores > 12, and Schirmer scores ≤ 3 mm were associated with a higher risk of high-frequency eye drop use. Susceptibility to environmental triggers could represent hyperalgesia/allodynia in high users. Patients with more than one of these predisposing factors should be informed of their risk and followed up more regularly to avoid medication overuse-associated complications. Punctal plugs, cyclosporine, intense pulsed light, and/or LipiFlow treatment could be conducted when indicated.

## Figures and Tables

**Table 1:** Multiple logistic regression models.

	Variables in the Equation	B	S.E.	*p*-Value	Odds Ratio (OR)
Model 1	SPEED	0.047	0.010	0.000	1.048
	OSDI	0.013	0.002	0.000	1.013
	LLT	−0.010	0.002	0.000	0.990
	MGE	−0.001	0.011	0.939	0.999
	Meiboscale (grade)	0.042	0.052	0.422	1.043
	Schirmer (mm)	−0.054	0.011	0.000	0.948
	TBUT (sec)	0.021	0.024	0.379	1.021
	SPK (grade)	0.666	0.048	0.000	1.947
	Bulbar redness (grade)	0.486	0.147	0.001	1.627
	Constant	−3.364	0.238	0.000	0.035
Model 2	SPEED	0.047	0.010	0.000	1.048
	OSDI	0.013	0.002	0.000	1.013
	LLT	−0.010	0.002	0.000	0.990
	Meiboscale (grade)	0.043	0.050	0.396	1.044
	Schirmer (mm)	−0.054	0.011	0.000	0.948
	TBUT (sec)	0.021	0.024	0.381	1.021
	SPK (grade)	0.666	0.048	0.000	1.947
	Bulbar redness (grade)	0.486	0.147	0.001	1.625
	Constant	−3.371	0.221	0.000	0.034
Model 3	SPEED	0.046	0.010	0.000	1.048
	OSDI	0.013	0.002	0.000	1.014
	LLT	−0.011	0.002	0.000	0.989
	Schirmer (mm)	−0.053	0.011	0.000	0.948
	TBUT (sec)	0.020	0.024	0.392	1.020
	SPK (grade)	0.671	0.047	0.000	1.955
	Bulbar redness (grade)	0.494	0.147	0.001	1.639
	Constant	−3.304	0.206	0.000	0.037
Model 4	SPEED	0.047	0.010	0.000	1.048
	OSDI	0.013	0.002	0.000	1.013
	LLT	−0.011	0.002	0.000	0.990
	Schirmer (mm)	−0.053	0.011	0.000	0.949
	SPK (grade)	0.664	0.047	0.000	1.943
	Bulbar redness (grade)	0.500	0.146	0.001	1.648
	Constant	−3.249	0.196	0.000	0.039

LLT: average lipid layer thickness; MGE: number of expressible meibomian gland expression; FTBUT: fluorescein tear-film break-up time; SPK: superficial punctate keratitis.

## Data Availability

The data presented in this study are available upon request from the corresponding author.

## Abbreviations

DED	dry eye disease
FTBUT	fluorescein tear-film break-up time
LLT	average lipid layer thickness
MGE	number of expressible meibomian gland expression
OSDI	Ocular Surface Disease Index
PB	number of partial blinks
PB rate (%)	partial blink rate
SPEED	Standardized Patient Evaluation of Eye Dryness
SPK	superficial punctate keratitis
TB	number of total blinks
